# Intraoperative radial nerve injury during coronary artery surgery – report of two cases

**DOI:** 10.1186/1749-7221-1-7

**Published:** 2006-12-05

**Authors:** Marianna Papadopoulou, Konstantinos Spengos, Apostolos Papapostolou, Georgios Tsivgoulis, Nikolaos Karandreas

**Affiliations:** 1 University of Athens School of Medicine, Department of Neurology, Eginition Hospital, Athens, Greece

## Abstract

**Background:**

Peripheral nerve injury and brachial plexopathy are known, though rare complications of coronary artery surgery. The ulnar nerve is most frequently affected, whereas radial nerve lesions are much less common accounting for only 3% of such intraoperative injuries.

**Case presentations:**

Two 52- and 50-year-old men underwent coronary artery surgery. On the first postoperative day they both complained of wrist drop on the left. Neurological examination revealed a paresis of the wrist and finger extensor muscles (0/5), and the brachioradialis (4/5) with hypoaesthesia on the radial aspect of the dorsum of the left hand. Both biceps and triceps reflexes were normoactive, whereas the brachioradialis reflex was diminished on the left. Muscles innervated from the median and ulnar nerve, as well as all muscles above the elbow were unaffected. Electrophysiological studies were performed 3 weeks later, when muscle power of the affected muscles had already begun to improve. Nerve conduction studies and needle electromyography revealed a partial conduction block of the radial nerve along the spiral groove, motor axonal loss distal to the site of the lesion and moderate impairment in recruitment with fibrillation potentials in radial innervated muscles below the elbow and normal findings in triceps and deltoid. Electrophysiology data pointed towards a radial nerve injury in the spiral groove. We assume external compression as the causative factor. The only apparatus attached to the patients' left upper arm was the sternal retractor, used for dissection of the internal mammary artery. Both patients were overweight and lying on the operating table for a considerable time might have caused the compression of their left upper arm on the self retractor's supporting column which was fixed to the table rail 5 cm above the left elbow joint, in the site where the radial nerve is directly apposed to the humerus.

**Conclusion:**

Although very uncommon, external compression due to the use of a self retractor during coronary artery surgery can affect – especially in obese subjects – the radial nerve within the spiral groove leading to paresis and should therefore be included in the list of possible mechanisms of radial nerve injury.

## Background

Peripheral nerve injury and brachial plexopathy are known, though rare complications of coronary artery surgery. The true incidence of nerve injury during general anesthesia remains unclear and probably is underestimated [1]. The ulnar nerve is most frequently affected accounting for one third of all nerve damages, whereas radial nerve lesions are much less common accounting for only 3% of such intraoperative injuries [2]. We report two cases of left radial nerve lesion during coronary artery surgery, presumably due to an external compression caused by a sternal retractor that is used for dissection of the internal mammary artery.

## Case presentations

### Case 1

A 52-year old obese man with known ischemic heart disease but no history of any neurological disease underwent coronary artery bypass surgery. Preoperative routinely performed diagnostic workup revealed no significant findings. During surgery he was laid supine on the operating table with both arms fully adducted to his side, fixed in the neutral position. Intraoperative monitoring included electrocardiography, pulse oxymetry and automatic blood pressure monitoring using a standard-size adult cuff affixed to the patient's right upper arm. No particular events occurred during anesthesia or surgery and recovery was good so that patient was transferred within a day from the intensive care unit to the normal ward.

However, on the first postoperative day he complained of wrist drop on the left. Neurological examination revealed a severe decrease in muscle power of the wrist and finger extensor muscles (0/5 MRC) and a slight brachioradialis paresis (4/5 MRC) accompanied by hypoaesthesia on the radial aspect of the dorsum mani. Biceps and triceps reflexes on the affected left arm were normoactive whereas the brachioradialis reflex was diminished. All muscles innervated from the median and ulnar nerve, as well as all the muscles above the elbow remained unaffected. The clinical diagnosis of radial nerve injury was set and rehabilitation therapy was recommended.

After hospital discharge and about three weeks after surgery the patient was referred for neurophysiological evaluation. In the meanwhile the extensor muscles had already begun to improve. Nerve conduction studies of both radial nerves were performed using surface electrodes. Compound muscle action potentials (CMAP) were recorded from the extensor digitorum communis muscle. The opposite radial nerve was examined for comparison. Supramaximal nerve stimulation was achieved by gradually increasing the stimulation power until the point where the amplitude of the waveform did no longer increased was reached. Electrical stimulation at the elbow, below and above the spiral groove, revealed an amplitude decline of the CMAP that was indicative of a partial conduction block of the left radial nerve along the spiral groove, whereas CMAP recordings of the right radial nerve were normal (Table 1). Moreover, motor axonal loss due to wallerian degeneration distal to the site of the lesion was suggested by the low distal CMAP. Needle electromyography enhanced this finding by revealing moderate impairment in recruitment with fibrillation potentials in radial innervated muscles below the elbow and normal findings in both triceps and deltoid muscles. The motor unit potentials were normal, a finding that is consistent with a recent nerve injury. In conclusion, all electrophysiological findings were indicative of a radial nerve injury in the spiral groove. The involvement of the brachioradialis muscle and the fact that both deltoid and triceps muscles remained unaffected practically excluded the differential diagnostic alternative of a posterior interosseus neuropathy and a posterior cord brachial plexus lesion respectively.

**Table 1:** Electrophysiological studies performed in both cases on radial nerves bilaterally indicative of a partial conduction block of the left radial nerve along the spiral groove with additional distal motor axonal loss due to wallerian degeneration.

Examination	Left side		Right side	
	Patient 1	Patient 2	Patient 1	Patient 2

CMAP-stimulation at the elbow	5 mV	4.5 mV	7.5 mV	8 mV
CMAP-stimulation below the spiral groove	4.5 mV	4.2 mV	7.2 mV	7.5 mV
CMAP-stimulation above the spiral groove	1.6 mV	1.3 mV	6.8 mV	7.0 mV
SNAP	15 μV	8 μV	63 μV	42 μV

CMAP stands for Compound Muscle Action Potentials.

SNAP stands for Sensory Nerve Action Potentials

### Case 2

Another 50-year-old obese man was referred for neurological and neurophysiological evaluation one month after having undergone coronary artery bypass surgery. He also reported suffering from a left wrist drop since the first postoperative day. Similarly to the previous case no incidents occurred during anaetshesia and surgery, during which exactly the same procedures were followed. Electromyography and nerve conduction studies were conducted and revealed identical findings suggestive of an injury of the left radial nerve in the spiral groove.

## Discussion

The similarity of these two cases is impressive. In both cases, there was no direct injury of the nerve during surgery; no neurotoxic material was injected; no event predisposing to nerve palsy (hypotension, hypoxia, electrolyte disturbances) occurred during or after anesthesia [3]; no malposition of the left arm on the operating table or later on the intensive care unit bed that may cause ischemic nerve injury was documented [4] and no stretch of the brachial plexus could have occurred [5], since the left arm was comfortably attached to the patients' body. Predisposing conditions such as arthritis or elbow instability were also excluded [6]. We therefore assume external compression as the causative factor.

The radial nerve is the largest nerve in the upper extremity, arising as an extension of the posterior cord of the brachial plexus. In the upper arm lies medially to the humerus, passes obliquely behind the humerus between the lateral and medial heads of the triceps and then enters the spiral groove to exit into the anterior compartment of the arm piercing the lateral intermuscular septum below the deltoid insertion. Then the nerve passes through the radial tunnel and divides into its terminal branches, the superficial radial, a pure sensory branch and posterior interosseus nerve, a pure motor branch. The most common cause of radial nerve injury is compression in the spiral groove which is a shallow groove formed deep to the lateral head of the triceps, where the nerve lies in close contact with the humerus. The radial nerve is compressed most often after piercing the lateral intermuscular ligament, where it lies unprotected by the triceps against the humerus. Patients with lesions of radial nerve in the spiral groove need to be differentiated from lesions of the posterior interosseus nerve and of the posterior cord of the brachial plexus. In the first case no sensory deficit is present and brachioradialis muscle escapes damage. In the second case, deltoid and triceps muscles are affected. Another differential diagnostic alternative that needs to be excluded is severe C7 and C8 radiculopathy that is characterized by a different sensory deficit (index, middle, ring and little finger) and a motor deficit in wrist flexion and forearm pronation as well. In both reported cases clinical and electrophysiological evidence establishes a radial nerve injury within the spiral groove. Finally, the differential diagnostic alternative of cerebral lesion imitating the clinical features of radial nerve palsy needs to be excluded. However in such a case the weakness is never limited solely to radial-innervated muscles and generally alterations in muscle tone and in the deep tendon reflexes of the limb are apparent. Moreover, when a patient with wrist drop caused by an upper motor neuron lesion grasps an object, involuntary synkinesis produces wrist extension as well. Since none of these features were present, central nervous system affection as cause of both cases of wrist drop could be clinically excluded.

Assuming an external compression as cause of such a lesion, we have to consider that the only apparatus attached to the patients' left upper arm was the sternal retractor, which is being used for the dissection of the internal mammary artery. Both patients were overweight and lying on the operating table for a considerable time might have caused the compression of their left upper arm on the self retractor's supporting column which is usually fixed to the table rail 5 cm above the left elbow joint, in the site where the radial nerve is unprotected directly apposed to the humerus.

Similar radial nerve compression has been attributed to an automatic blood pressure monitoring cuff [7] and a Kent retractor used for upper abdominal surgery [8]. There have been only three further reports of radial nerve palsy due to the use of a self retractor for the dissection of the left internal mammary artery for coronary artery surgery [9-11]. Similarly to our cases where symptoms ceased within two months, in all reported cases the lesion was reversible.

Transient neurologic symptoms result from action potential propagation failure caused by ischemia. The most widely used classification of peripheral nerve injury is the one introduced by Seddon and Sunderland [12,13]. Focal pressure, when brief and modest, distorts the myelin producing segmental conduction block without wallerian degeneration. This is termed neurapraxia. With increasing pressure, the axon is interrupted, resulting in secondary wallerian degeneration distally. If supporting structures, e.g. basal lamina and Schwann cells, remain intact this injury is termed axonotmesis. Severe injury that results in complete disruption of the nerve and all the supporting structures is termed neurotmesis. Conduction block is reversible whereas wallerian degeneration and axonal loss may have a poorer prognosis with slow and incomplete recovery [14]. Wallerian degeneration is completed within 7–10 days. Spontaneous activity, generated by denervated muscles, appears approximately during the second week, first proximally and then more distally. It becomes widespread after the third week and is most prominent after the fourth week. Thus repeated neurophysiological studies are needed to confirm the diagnosis and follow the process of reinnervation.

Although very uncommon, external compression due to the use of a self retractor during coronary artery surgery can cause – especially in obese subjects – radial nerve palsy and should probably be included in the list of possible mechanisms of radial nerve injury. Considering the small number of reported similar cases and the fact that symptoms are reversible, it could be assumed that the frequency of such intraoperative complications is probably underestimated. Prospective studies or even retrospective evaluation might be helpful in order to estimate the true incidence of intaoperative nerve injuries, understand the causative mechanism and eventually find effective preventing strategies.

## Competing interests

The author(s) declare that they have no competing interests.

## Authors' contributions

MP performed in both cases the electromyographic studies in both cases and drafted the manuscript together with KS, who also made the appropriate literature review. AP performed the conduction studies whereas GT examined clinically both patients. NK coordinated the work for this paper and also helped drafting the manuscript with his critical remarks. All authors read and approved the final manuscript.

## References

[B1] Sawyer RJ Richmond MN Hickey JD Jarrratt JA Peripheral nerve injuries associated with anaesthesia Anaesthesia 2000 1 980 991 10.1046/j.1365-2044.2000.01614.x 11012494

[B2] Kroll DA Caplan RA Posner K Ward RJ Cheney FW Nerve injury associated with anesthesia Anesthesiology 1990 1 202 207 10.1097/00000542-199008000-00002 2382845

[B3] Cheney FW Domino KB Caplan RA Posner KL Nerve injury associated with anesthesia Anesthesiology 1999 1 1062 1069 10.1097/00000542-199904000-00020 10201678

[B4] Dawson DM Krarup C Perioperative nerve lesions Arch Neurol 1989 1 1355 1360 2556097 10.1001/archneur.1989.00520480099027

[B5] Clausen EG Postoperative anesthetic paralysis of the brachial plexus Surgery 1942 1 933 941

[B6] Tuncali BE Tuncali B Kuvaki B Cinar O Doğan A Elar Z Radial nerve injury after general anaesthesia in the lateral decubitus position Anaesthesia 2005 1 602 604 10.1111/j.1365-2044.2005.04177.x 15918832

[B7] Lin CC Jawan B de Villa MV Chen FC Liu PP Blood pressure cuff compression injury of the radial nerve J Clin Anesth 2001 1 306 308 10.1016/S0952-8180(01)00262-8 11435057

[B8] Lee HC Kim HD Park WK Rhee HD Kim KJ Radial nerve paralysis due to Kent retractor during upper abdominal operation Yonsei Med J 2003 1 1106 1109 14703626 10.3349/ymj.2003.44.6.1106

[B9] Guzman F Naik S Weldon OG Hilton CJ Transient radial nerve injury related to the use of a self retaining retractor for internal mammary artery dissection J Cardiovasc Surg 1989 1 1015 1016 2600114

[B10] Fernandez de Caleya D Duarte J Lozano A Torrente N Radial nerve injury by external compression during the dissection of the internal mammary artery in coronary surgery Rev Esp Anestesiol Reanim 1992 1 371 373 1293655

[B11] Briffa NP Price C Grotte GJ Keenan DJ Radial nerve injury in patients undergoing coronary artery bypass grafting Ann Thorac Surg 1992 1 1149 1150 1596151 10.1016/0003-4975(92)90419-5

[B12] Seddon H Three types of nerve injury Brain 1943 1 237 288

[B13] Sunderland S Nerve injuries and their repair, a critical appraisal 1991 Edinburgh, Churchill Livingstone

[B14] Fowler TJ Danta G Gilliatt RW Recovery of nerve conduction after a pneumatic tourniquet: observation on the hint-limb of the balloon J Neurol Neurosurg Psychiatry 1972 1 638 647 4628467 10.1136/jnnp.35.5.638 PMC494143

